# Disproportionate Vulnerability to and Unique Aggregation Pattern of Non-AIDS Comorbidities Among Women With HIV in China

**DOI:** 10.1093/ofid/ofaf046

**Published:** 2025-01-29

**Authors:** Xiaoxiao Chen, Congcong Guo, Tingting Wang, Weiwei Shen, Shanling Wang, Yating Wang, Tailin Chen, Miaochen Wang, Haijiang Lin, Na He

**Affiliations:** Department of Epidemiology, School of Public Health, and Key Laboratory of Public Health Safety of the Ministry of Education, Fudan University, Shanghai, China; Taizhou City Center for Disease Control and Prevention, Taizhou, China; Taizhou Central Blood Station, Taizhou, China; Jiaojiang District Center for Disease Control and Prevention, Taizhou, China; Taizhou City Center for Disease Control and Prevention, Taizhou, China; Taizhou City Center for Disease Control and Prevention, Taizhou, China; Taizhou City Center for Disease Control and Prevention, Taizhou, China; Taizhou City Center for Disease Control and Prevention, Taizhou, China; Department of Epidemiology, School of Public Health, and Key Laboratory of Public Health Safety of the Ministry of Education, Fudan University, Shanghai, China; Yi-Wu Research Institute, Fudan University, Shanghai, China; Department of Epidemiology, School of Public Health, and Key Laboratory of Public Health Safety of the Ministry of Education, Fudan University, Shanghai, China; Yi-Wu Research Institute, Fudan University, Shanghai, China; Taizhou City Center for Disease Control and Prevention, Taizhou, China; Department of Epidemiology, School of Public Health, and Key Laboratory of Public Health Safety of the Ministry of Education, Fudan University, Shanghai, China; Yi-Wu Research Institute, Fudan University, Shanghai, China; Shanghai Institute of Infectious Diseases and Biosecurity, Fudan University, Shanghai, China

**Keywords:** age, comorbidity, HIV, multimorbidity, sex

## Abstract

**Background:**

Whether and how sex plays differential roles in aging-related multimorbidity among people with HIV (PWH) is poorly characterized.

**Methods:**

We included 2479 PWH and 5376 people without HIV from the baseline assessment of the CHART cohort (Comparative HIV and Aging Research in Taizhou). Ten non-AIDS comorbidities were investigated. Multiple logistic regression was used to assess the correlates of multimorbidity, defined as the coexistence of ≥2 non-AIDS comorbidities. Multimorbidity patterns were identified through hierarchical cluster analysis.

**Results:**

The prevalence of multimorbidity was higher in PWH than in people without HIV (74.6% vs 66.9%, *P* < .001). This difference was particularly pronounced in women in each age group from 18 through 59 years and among men in each age group from 18 through 49 years. A significant interaction between sex and HIV on multimorbidity was identified (*P* < .001), with the strength of the association between HIV infection and multimorbidity being stronger in women than in men. Women with HIV presented a unique aggregation pattern of multimorbidity, where neuropsychiatric disorders (depression, neurocognitive impairment) clustered with cardiometabolic diseases. In contrast, all men and women without HIV manifested a similar multimorbidity pattern, where depression and neurocognitive impairment were clustered with hematologic abnormalities but not with cardiometabolic diseases.

**Conclusions:**

Earlier onset and higher burden of multimorbidity in PWH, as well as disproportionate vulnerability to and a unique multimorbidity pattern among women with HIV, underscore the urgent need for early and sexually oriented integrative interventions and health services targeting multimorbidity in PWH.

## Teaser

Women with HIV were at a disproportionally high risk of living with multimorbidity at younger ages and manifested a unique multimorbidity pattern with neuropsychiatric disorders or mental health conditions clustered with cardiometabolic diseases.

The widespread use of combined antiretroviral therapy (cART) has resulted in improved life expectancy for people with HIV (PWH), but it also increases the risk of aging-related health conditions [1]. Chronic immunodeficiency and inflammation caused by HIV [2], the toxic effects of cART [3], and a high prevalence of behavior risk factors [4] are implicated in the earlier onset and higher burden of non-AIDS comorbidities (NACMs) among PWH as compared with people without HIV (PWoH). Increasing evidence suggests that women with HIV experience a significantly higher prevalence and incidence of NACM than men with HIV [5–8].

Multimorbidity, defined as the coexistence of at least 2 health conditions in the same individual [9], is strongly associated with age, with prevalence in general geriatric populations ranging from 55% to 98% [10]. Several studies have revealed a higher prevalence of multimorbidity in PWH as compared with the general population [11, 12]. In the context of HIV infection, multimorbidity is associated with polypharmacy, diminished quality of life, and increasing mortality [13–15]. A significant increase in mortality attributed to non–AIDS-related causes has been observed in PWH following the widespread adoption of cART [16, 17]. However, while several studies have explored multimorbidity patterns in PWH by focusing on cardiovascular and metabolic diseases, mental health problems, and lifestyle behaviors [18, 19], none have examined sex differences in multimorbidity patterns among PWH. To inform sex-tailored health care strategies for managing multimorbidity in HIV infection, we investigated sex disparities in the prevalence, patterns, and associated factors of multimorbidity in participants of the CHART cohort in China (Comparative HIV and Aging Research in Taizhou) [20].

## METHODS

### Study Design and Participants

CHART is an ongoing prospective cohort study investigating HIV and aging-related comorbidities among PWH and PWoH in Zhejiang province, eastern China [20]. PWH aged ≥18 years were recruited during routine follow-up visits at local Centers for Disease Control and Prevention and were registered in China's HIV/AIDS Comprehensive Response Information Management System. Concurrently, PWoH were purposively selected from 6 communities (Yuhuan, Luqiao, Tiantai, Huangyan, Linhai, and Sanmen), with confirmation of HIV seronegativity through testing. A total of 2892 PWH and 5415 age- and sex-matched community residents without HIV were enrolled and completed a comprehensive baseline assessment between January 2017 and December 2019. For the present study, 2479 PWH and 5376 PWoH with complete data on multimorbidity were included in the analysis. The percentages of missing data for key NACM variables were as follows: 1.6%, abnormal liver function (ALF); 1.7%, anemia; 0.1%, depression; 1.7%, diabetes; 1.6%, dyslipidemia; 2.8%, electrocardiographic abnormality (EA); 0.4%, hypertension; 0.2%, neurocognitive impairment (NCI); 1.6%, renal impairment (RI); and 2.5%, subclinical atherosclerosis (SCA).

### Data Collection

All participants were asked to complete a standardized questionnaire to assess demographics (sex assigned at birth, age, marital status, and education level), lifestyle risk factors (cigarette use, alcohol use, and exercise), neurocognitive performances per the Chinese version of the Mini-mental State Examination [21], depression symptoms, and history of diseases. Physical examinations, blood pressure measurements, hemoglobin A1c tests, and electrocardiogram measurements were also conducted. Laboratory tests were performed with serum samples obtained after at least 8 hours of fasting, within 1 day of collection, to assess serum markers, including alanine transaminase, aspartate aminotransferase, total cholesterol, low-density lipoprotein cholesterol, triglycerides, and serum creatine. Carotid intima-media thickness was measured as previously described [20]. HIV-related information, including HIV transmission mode, CD4 cell count, and cART initiation and regimen, was extracted from the Chinese HIV/AIDS Comprehensive Response Information Management System.

Current cigarette use was defined as having smoked within the past month. Alcohol use was defined as drinking regularly or daily within the past month. Exercise behavior was defined as exercising at least 3 times a week. Body mass index (BMI) was calculated as weight in kilograms divided by the square of height in meters, and waist-to-hip ratio (WHR) was calculated by dividing the waist circumference (centimeters) by hip circumference (centimeters). BMI was categorized into underweight (<18.5 kg/m^2^), normal weight (18.5–23.9 kg/m^2^), and overweight/obesity (≥24 kg/m^2^) [22]. A high WHR, indicative of abdominal obesity, was defined as ≥0.90 for men and ≥0.85 for women.

### Outcome Measures

In this study, the primary outcome was multimorbidity, defined as the coexistence of ≥2 of the following 10 NACMs or health conditions: ALF, anemia, depression, diabetes, dyslipidemia, EA, hypertension, NCI, RI, and SCA. ALF was diagnosed when alanine transaminase or aspartate aminotransferase levels exceeded the upper limit of normal for local reference laboratories [23]. Specifically, ALF was defined as alanine transaminase >50 U/L in men or >40 U/L in women and aspartate aminotransferase >40 U/L in men or >35 U/L in women. Anemia was defined as a hemoglobin concentration <120 g/L in men or <110 g/L in women [24]. Diabetes was defined as a hemoglobin A1c ≥6.5% or a previous clinical diagnosis of diabetes [25]. Dyslipidemia was defined as total cholesterol ≥5.2 mmol/L, low-density lipoprotein cholesterol ≥3.4 mmol/L, or triglycerides ≥1.7 mmol/L [26]. EA was defined as tachycardia, bradycardia, ST/T wave abnormalities, left ventricle hypertrophy, axis deviation, or other changes based on Minnesota codes [27]. Hypertension was defined as a systolic blood pressure ≥140 mm Hg, diastolic blood pressure ≥90 mm Hg, or a previous clinical diagnosis of hypertension [28]. SCA was defined as a carotid intima-media thickness ≥780 µm [29]. RI was defined as an estimated glomerular filtration rate <90/mL/min/1.73 m^2^ [30]. NCI was defined by the Mini-mental State Examination, with thresholds based on educational level: ≤17 for individuals with no formal education, ≤20 for those with primary school education (≤6 years), and ≤24 for those with junior school graduates or above (≥7 years) [31]. Depression was measured with the 9-item version of the Zung Self-rating Depression Scale and defined as a score ≥18 [31, 32]. The Mini-mental State Examination and Zung Self-rating Depression Scale are widely used screening instruments for NCI [31, 33] and depression [34] among Chinese PWH, respectively.

### Statistical Analysis

Analyses were performed with R software version 4.3.0. *P* < .05 was considered statistically significant. Group comparisons were conducted by Wilcoxon rank sum tests for continuous variables and χ^2^ tests for categorical variables. Multiple logistic regression was performed to analyze the associates and interaction term (HIV × sex) on the multiplicative scale of multimorbidity prevalence. The adjusted relative excess risk due to interaction and the corresponding 95% CI were calculated to assess the additive interaction of HIV and sex on multimorbidity [35]. Marital status was excluded from the regression model due to collinearity, as indicated by variance inflation factor [36]. Linear regression was used to examine the correlates of NACM burden, defined as the total number of NACMs per participant.

Hierarchical cluster analysis was employed to identify multimorbidity patterns, following these steps: (1) The presence or absence of each NACM was encoded as binary data (1 for presence, 0 for absence). (2) A dissimilarity matrix was constructed via the binary method to calculate the pairwise proximity between NACMs. (3) The Ward method was then applied to the dissimilarity matrix to perform agglomerative hierarchical clustering, which minimizes the within-cluster variance. (4) A dendrogram was generated to visualize the clustering of NACMs, revealing their hierarchical relationships and the patterns of multimorbidity in the study population.

## RESULTS

### Participant Characteristics

Among the 7855 study participants, 2060 (26.2%) were women (547 HIV positive, 1513 HIV negative) and 5795 (73.8%) were men (1932 HIV positive, 3863 HIV negative). The median (IQR) age was 44.0 years (32.0–55.0). The majority were married and had limited education (Table 1). Characteristics such as marital status, education, cigarette use, exercise, BMI, and WHR differed significantly by sex and HIV infection status.

**Table 1.** Characteristics of Study Participants

Characteristic	Total (n = 7855)^a^	Women With HIV (n = 547)	Women Without HIV (n = 1513)	*P* Value^b^	Men With HIV (n = 1932)	Men Without HIV (n = 3863)	*P* Value ^b^
Age group, y				.122			.193
18–29	1490 (19.0)	73 (13.3)	265 (17.5)		411 (21.3)	741 (19.2)	
30–39	1689 (21.5)	137 (25.0)	348 (23.0)		377 (19.5)	827 (21.4)	
40–49	1822 (23.2)	117 (21.4)	336 (22.2)		475 (24.6)	894 (23.1)	
50–59	1389 (17.7)	112 (20.5)	323 (21.3)		312 (16.1)	642 (16.6)	
60–69	1010 (12.9)	86 (15.7)	191 (12.6)		237 (12.3)	496 (12.8)	
≥70	455 (5.8)	22 (4.0)	50 (3.3)		120 (6.2)	263 (6.8)	
Married	5656 (72.0)	402 (73.5)	1278 (84.5)	<.001	1040 (53.8)	2936 (76.0)	<.001
Education				<.001			<.001
Primary school or less	2715 (34.6)	315 (57.6)	581 (38.4)		649 (33.6)	1170 (30.3)	
Middle school	2433 (31.0)	170 (31.1)	388 (25.6)		665 (34.4)	1210 (31.3)	
High school or above	2707 (34.5)	62 (11.3)	544 (36.0)		618 (32.0)	1483 (38.4)	
Current cigarette use				<.001			<.001
No	5358 (68.3)	523 (95.6)	1505 (99.5)		1270 (65.7)	2060 (53.4)	
Yes	2491 (31.7)	24 (4.4)	8 (0.5)		662 (34.3)	1797 (46.6)	
Alcohol use	1278 (16.3)	12 (2.2)	45 (3.0)	.342	209 (10.8)	1012 (26.3)	<.001
Exercise	2527 (32.2)	123 (22.5)	444 (29.3)	.002	716 (37.1)	1244 (32.2)	<.001
BMI, kg/m^2^, median (IQR)	23.2 (20.9–25.7)	21.7 (19.8–23.9)	23.0 (20.9–25.6)	<.001	21.9 (20.0–23.9)	24.3 (21.9–26.7)	<.001
BMI, kg/m^2^				<.001			<.001
<18.5	524 (6.7)	69 (12.6)	112 (7.4)		168 (8.7)	175 (4.5)	
18.5–23.9	4067 (51.8)	343 (62.8)	799 (52.8)		1296 (67.2)	1629 (42.2)	
≥24	3259 (41.5)	134 (24.5)	602 (39.8)		466 (24.1)	2057 (53.3)	
WHR, median (IQR)	0.89 (0.84–0.93)	0.88 (0.84–0.92)	0.84 (0.79–0.89)	<.001	0.89 (0.86–0.92)	0.90 (0.86–0.94)	<.001
High WHR	3811 (48.5)	369 (67.6)	696 (46.0)	<.001	857 (44.4)	1889 (48.9)	.001
Years with HIV diagnosis							
<3	1615 (65.3)	308 (56.4)	…		1307 (67.8)	…	
≥3	858 (34.7)	238 (43.6)	…		620 (32.2)	…	
HIV transmission mode							
Heterosexual	1663 (67.3)	528 (97.1)	…		1135 (58.9)	…	
Homosexual	767 (31.0)	0 (0.0)	…		767 (39.8)	…	
Other ^c^	41 (1.7)	16 (2.9)	…		25 (1.3)	…	
Years on cART							
cART naive	531 (21.5)	110 (20.2)	…		421 (21.9)	…	
<3	1255 (50.8)	249 (45.8)	…		1006 (52.2)	…	
≥3	684 (27.7)	185 (34.0)	…		499 (25.9)	…	
CD4 cell count, cells/μL							
<200	448 (18.2)	69 (12.7)	…		379 (19.7)	…	
200–349	632 (25.6)	119 (21.8)	…		513 (26.7)	…	
≥350	1387 (56.2)	357 (65.5)	…		1030 (53.6)	…	
Hypertension	2314 (29.5)	112 (20.5)	383 (25.3)	.023	437 (22.6)	1382 (35.8)	<.001
Diabetes	761 (9.7)	39 (7.1)	109 (7.2)	.954	140 (7.2)	473 (12.2)	<.001
Dyslipidemia	5004 (63.7)	301 (55.0)	880 (58.2)	.204	1091 (56.5)	2732 (70.7)	<.001
SCA	2409 (30.7)	181 (33.1)	306 (20.2)	<.001	706 (36.5)	1216 (31.5)	<.001
RI	2366 (30.1)	181 (33.1)	453 (29.9)	.171	520 (26.9)	1212 (31.4)	<.001
NCI	573 (7.3)	101 (18.5)	73 (4.8)	<.001	231 (12.0)	168 (4.3)	<.001
Depression	1908 (24.3)	251 (45.9)	301 (19.9)	<.001	755 (39.1)	601 (15.6)	<.001
Anemia	603 (7.7)	90 (16.5)	177 (11.7)	.005	217 (11.2)	119 (3.1)	<.001
ALF	372 (4.7)	41 (7.5)	43 (2.8)	<.001	103 (5.3)	185 (4.8)	.371
EA	3015 (38.4)	220 (40.2)	506 (33.4)	.004	763 (39.5)	1526 (39.5)	.994

Data are presented as No. (%) unless noted otherwise.

Abbreviations: ALF, abnormal liver function; BMI, body mass index; cART, combination antiretroviral therapy; EA, electrocardiographic abnormality; NCI, neurocognitive impairment; RI, renal impairment; SCA, subclinical atherosclerosis; WHR, waist-to-hip ratio.

^a^Number of participants with missing information: marital status (2), cigarette use (6), alcohol use (16), exercise (5), BMI (5), high WHR (3), time on cART (9), time since HIV diagnosis (6), HIV transmission mode (8), CD4 cell count (12).

^b^χ^2^ test performed for categorical variables and Wilcoxon rank sum test for continuous variables.

^c^Injection drug use and blood transfusion.

For PWH, the median (IQR) time since HIV diagnosis was 1.17 years (0.18–4.40). Of these, 67.3% were infected through heterosexual contact, whereas 37.0% were homosexually infected. Additionally, 78.5% were undergoing cART, and the median (IQR) CD4 cell count was 390 (243–540) cells/μL, with 56.2% having a CD4 count >350 cells/μL (Table 1). When compared with men with HIV, women with HIV had a longer time with HIV diagnosis (χ^2^ = 24.5, *P* < .001), a higher proportion of heterosexual transmission (χ^2^ = 315.2, *P* < .001), longer time with cART (χ^2^ = 14.0, *P* < .001), and higher CD4 cell count (χ^2^ = 26.3, *P* < .001).

### Prevalence and Associates of Multimorbidity

#### Age- and Sex-Specific Prevalence of Multimorbidity in PWH vs PWoH

**Figure 1.**
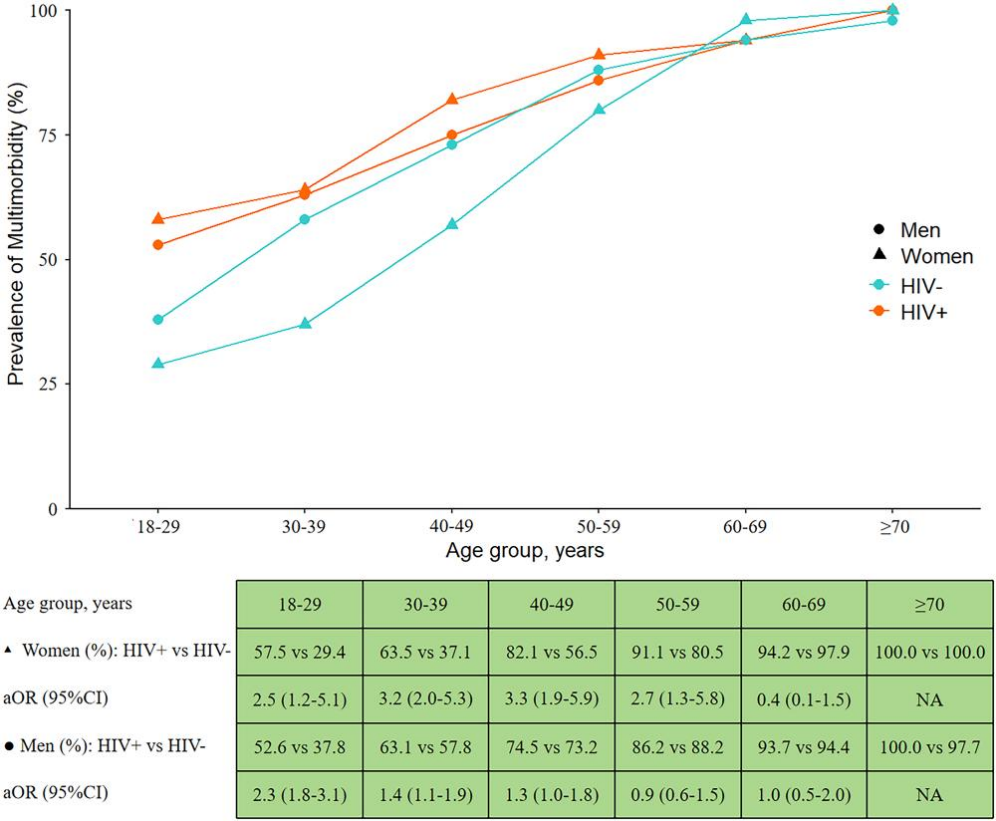
Prevalence of multimorbidity by sex, HIV status, and age group. Adjusted odds ratio (aOR) and 95% CI for the association of HIV infection with multimorbidity were obtained by multivariable logistic regression analysis after adjusting for education, cigarette use, alcohol use, exercise, body mass index, and waist-to-hip ratio across age groups (18–29, 30–39, 40–49, 50–59, 60–69, ≥70 years) and sex (women, men).

Overall the prevalence of multimorbidity was significantly higher in PWH than PWoH (74.6% vs 66.9%, *P* < .001; Supplementary Table 1). Figure 1 illustrates the prevalence of multimorbidity by sex, HIV status, and age group, as well as the results of multivariable logistic regression analyses comparing age- and sex-specific prevalence of multimorbidity between PWH and PWoH. Among women, those who were HIV positive had a significantly higher prevalence of multimorbidity than their counterparts who were HIV negative in each age group from 18 to 59 years. Among men, those who were HIV positive also had a significantly higher prevalence of multimorbidity than their counterparts who were HIV negative in each age group from 18 to 49 years, with relatively lower strength (adjusted odds ratio) of the age-specific association between HIV infection and multimorbidity in men than women. There was no significant difference in the prevalence of multimorbidity between the HIV statuses (positive and negative) for women aged ≥60 years and men ≥50 years. For most demographic and lifestyle subgroups, the prevalence of multimorbidity was highest among women with HIV, followed by men with HIV, men without HIV, and women without HIV, in decreasing order (Supplementary Figure 1).

Multiple logistic regression adjusting for age, education, cigarette use, alcohol use, exercise, BMI, and WHR revealed a significant interaction between HIV and sex on multimorbidity. The adjusted odds ratio for the interaction term on the multiplicative scale was 1.8 (95% CI, 1.3–2.4; *P* < .001), suggesting that the strength of the association between HIV and multimorbidity was stronger in women vs men. On the additive scale, the adjusted relative excess risk due to interaction was 0.5 (95% CI, .3–.7; *P* < .001), indicating that the combined effect of HIV and female sex on multimorbidity was greater than the sum of their individual effects (Supplementary Table 2).

#### Associates of Multimorbidity

As shown in Table 2, multiple logistic regression analyses revealed that multimorbidity was positively associated with age, BMI ≥24 kg/m^2^ and high WHR among all participants, as well as within women and men. Higher education was negatively associated with multimorbidity among all participants and men but not women.

**Table 2.** Multivariable Logistic Regression Analyses of Correlates With Multimorbidity

	All Participants			Women			Men		
Variable	Multimorbidity, No. (%)	aOR (95% CI)	*P* Value^a^	Multimorbidity, No. (%)	aOR (95% CI)	*P* Value	Multimorbidity, No. (%)	aOR (95% CI)	*P* Value
HIV status									
Negative	3597 (66.9)	1 [Reference]		894 (59.1)	1 [Reference]		2703 (70.0)	1 [Reference]	
Positive	1849 (74.6)	1.7 (1.5–2.0)	<.001	430 (78.6)	2.7 (2.0–3.6)	<.001	1419 (73.4)	1.5 (1.3–1.8)	<.001
Sex									
Men	4122 (71.1)	1 [Reference]		…	…	…	…	…	…
Women	1324 (64.3)	0.7 (.6–.8)	<.001	…	…	…	…	…	…
Age group, y									
18–29	616 (41.3)	1 [Reference]		120 (35.5)	1 [Reference]		496 (43.1)	1 [Reference]	
30–39	932 (55.2)	1.5 (1.3–1.8)	<.001	216 (44.5)	1.3 (.9–1.7)	.144	716 (59.5)	1.6 (1.4–2.0)	<.001
40–49	1294 (71.0)	2.6 (2.2–3.0)	<.001	286 (63.1)	2.4 (1.7–3.4)	<.001	1008 (73.6)	2.7 (2.2–3.3)	<.001
50–59	1197 (86.2)	6.6 (5.3–8.1)	<.001	362 (83.2)	6.6 (4.5–9.9)	<.001	835 (87.5)	6.8 (5.3–8.8)	<.001
60–69	958 (94.9)	17.2 (12.4–23.9)	<.001	268 (96.8)	35.8 (16.8–75.9)	<.001	690 (94.1)	13.9 (9.7–20.1)	<.001
≥70	449 (98.7)	70.8 (31.1–161.4)	<.001	72 (100.0)	…	…	377 (98.4)	56.6 (24.6–130)	<.001
Education									
Primary school or less	2326 (85.7)	1 [Reference]		734 (81.9)	1 [Reference]		1592 (87.5)	1 [Reference]	
Middle school	1694 (69.6)	0.8 (.7–1.0)	.011	341 (61.1)	0.9 (.7–1.2)	.470	1353 (72.2)	0.8 (.6–1.0)	.019
High school or above	1426 (52.7)	0.7 (.6–.9)	<.001	249 (41.1)	0.8 (.6–1.1)	.136	1177 (56.0)	0.7 (.6–.9)	.001
Cigarette use									
Past or never	3663 (68.4)	1 [Reference]		1304 (64.3)	1 [Reference]		2359 (70.8)	1 [Reference]	
Current	1777 (71.3)	1.0 (.9–1.2)	.487	20 (62.5)	0.8 (.4–1.9)	.632	1757 (71.5)	1.0 (.9–1.2)	.678
Alcohol use									
No	4441 (67.7)	1 [Reference]		1280 (64.0)	1 [Reference]		3161 (69.3)	1 [Reference]	
Yes	990 (77.5)	1.0 (.9–1.2)	.680	42 (73.7)	1.1 (.6–2.1)	.819	948 (77.6)	1.0 (.8–1.2)	.931
Exercise									
No	3646 (68.5)	1 [Reference]		966 (64.7)	1 [Reference]		2680 (70.0)	1 [Reference]	
Yes	1798 (71.2)	1.0 (.9–1.2)	.463	358 (63.1)	1.0 (.8–1.3)	.745	1440 (73.5)	1.1 (.9–1.2)	.395
BMI, kg/m^2^									
<18.5	275 (52.5)	0.8 (.7–1.0)	.062	92 (50.8)	0.8 (.6–1.2)	.327	183 (53.4)	0.8 (.6–1.0)	.078
18.5–23.9	2610 (64.2)	1 [Reference]		684 (59.9)	1 [Reference]		1926 (65.8)	1 [Reference]	
≥24	2558 (78.5)	1.8 (1.6–2.0)	<.001	547 (74.3)	1.4 (1.1–1.8)	.010	2011 (79.7)	2.0 (1.7–2.3)	<.001
High WHR									
No	2398 (59.3)	1 [Reference]		508 (51.1)	1 [Reference]		1890 (62.0)	1 [Reference]	
Yes	3046 (79.9)	1.4 (1.2–1.6)	<.001	815 (76.5)	1.3 (1.0–1.6)	.041	2231 (81.2)	1.4 (1.2–1.6)	<.001

Abbreviations: aOR, adjusted odds ratio; BMI, body mass index; WHR, waist-to-hip ratio.

^a^Interaction item (HIV × sex) was statistically significant (*P* < .001 for interaction) in the adjusted logistic regression model including all variables listed in the table.

### Burden of NACM

Supplementary Figure 2 showed the burden and distribution of NACM by sex, HIV status, and age group. Overall the mean NACM burden was significantly higher in PWH vs PWoH (2.6 vs 2.4, *P* < .001). PWH had a significantly higher age-stratified NACM burden than PWoH from ages 18 to 59 years among women and from 18 to 49 years among men. There were no significant differences in NACM burden between PWH and PWoH in women ≥60 years or men ≥50 years. Furthermore, NACM burden was positively associated with lower education, BMI ≥24 kg/m^2^, and high WHR and, in men only, exercise (Supplementary Table 3).

### Multimorbidity Patterns

Supplementary Figure 3 presents the most prevalent combinations of multimorbidity by sex and HIV status. The number of multimorbidity combinations with a prevalence >1% were 18 for women with HIV, 15 for women without HIV, 13 for men with HIV, and 16 for men without HIV. Among women with HIV, the 3 most prevalent multimorbidity combinations were dyslipidemia-depression, dyslipidemia-EA, and dyslipidemia-RI-depression. Among men with HIV, the most prevalent combination was dyslipidemia-depression. For women and men without HIV, the most common combination was dyslipidemia-EA.

**Figure 2.**
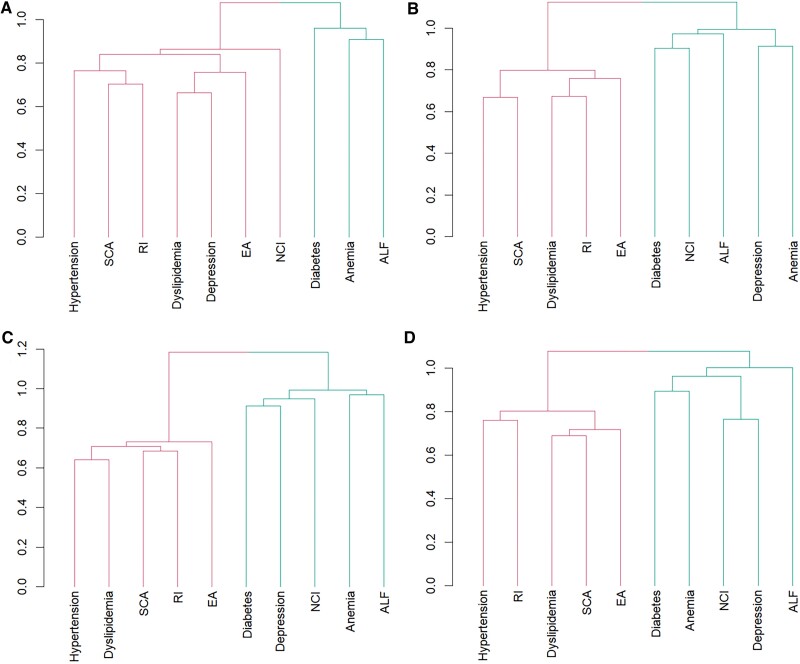
Dendrograms of cluster analysis show the unique aggregation pattern of comorbid health conditions in (*A*) women with HIV as compared with the uniform patterns of (*B*) women without HIV, (*C*) men with HIV, and (*D*) men without HIV. ALF, abnormal liver function; EA, electrocardiographic abnormality; NCI, neurocognitive impairment; RI, renal impairment; SCA, subclinical atherosclerosis.

Figure 2 shows the multimorbidity patterns across HIV infection status and sex. Women with HIV presented a unique multimorbidity pattern, with neuropsychiatric disorders or mental health conditions (depression, NCI) clustering with cardiometabolic diseases (hypertension, dyslipidemia, SCA, RI, and EA; Figure 2*A*). In contrast, all other groups including men with or without HIV and women without HIV manifested a similar multimorbidity pattern, where neuropsychiatric disorders or mental health conditions (depression, NCI) were clustered with hematologic abnormalities (hemoglobin A1c–defined diabetes, anemia, ALF) but not cardiometabolic diseases (Figure 2*B–D*).

## DISCUSSION

To the best of our knowledge, this is the first study designed to delineate and compare the epidemiologic characteristics of NACM and multimorbidity pattern by HIV infection status, sex, and age in Chinese population. We found that while women without HIV had the lowest prevalence of multimorbidity, women with HIV were disproportionally at higher risk of living with multimorbidity. This observation is consistent with recent studies, primarily conducted in the United States, showing that women with HIV have a significantly higher prevalence and incidence of age-related NACM as compared with men with HIV [6, 37–39] and that the burden of NACM is higher among women with HIV than women without HIV [7, 40]. The significant interaction between sex and HIV infection suggests potential sex-linked differences in the epidemiology and pathogenesis of NACM in PWH, which warrants further investigation. Furthermore, the largest gap in age- and sex-specific prevalence of multimorbidity between PWH and PWoH was observed in women at younger ages, suggesting that women with HIV may experience earlier onset or higher vulnerability to NACM. This earlier onset and higher prevalence of NACM in women with HIV could lead to a greater burden of morbidity [41]. These observations highlight the need for further research into the sex-specific aspects of NACM in PWH, which could inform the development of tailored, sexually differentiated health interventions.

Age was the most important determinant of aging-related NACMs. In this study, the prevalence and association strength of non-AIDS multimorbidity strikingly increased with age, independent of HIV status, demographics, and lifestyle factors. Nevertheless, the earlier onset and higher burden of multimorbidity among PWH reiterate the importance of early intervention for NACM in these population. As expected, high BMI and high WHR were significantly associated with a higher prevalence of multimorbidity, as both are well-recognized risk factors for various NACMs. This association may be particularly relevant for PWH, who are at increased risk for overweight/obesity and abdominal obesity due to the side effects of antiretroviral drugs [42].

We, for the first time, identified that women with HIV manifested a unique multimorbidity pattern in which neuropsychiatric disorders or mental health conditions were clustered with cardiometabolic diseases. This unique pattern suggests that female sex may play a significant role in shaping the risks and pathogenesis of NACM, including neuropsychiatric disorders, although the mechanisms underlying this clustering and the role of female sex in shaping immune responses to HIV and the pathogenesis of NACM remain unclear and warrant further longitudinal investigation. Previous studies have reported that women have a greater risk of NCI than men [43]. A global survey found that 7 of 10 women with HIV report depression and other mental health issues, with >50% experiencing multiple issues [44]. Stigma that targeted women's identities and social marginalization are critical reasons for the increased mental health issues after HIV diagnosis [44]. It is widely recognized that women have higher levels of immune activation and inflammation in response to HIV infection than men [45, 46]. Further research is needed to clarify the relationship between disproportionate immune activation/inflammation and the clustering of cardiometabolic diseases and mental health conditions in women with HIV. Other possible mechanisms underlying sex differences in NACM risks and multimorbidity pattern may include the effects of sex hormones during the menopausal transition [47], differing pharmacokinetic profiles [48], and sexual differences in gut permeability leading to microbial translocation [49].

Our study had several limitations. First, this was a cross-sectional survey, and only the prevalence of NACM and multimorbidity was assessed. Therefore, our ability to make causal inferences was limited. However, the major findings regarding the roles of HIV status, age, and sex in the risks and patterns of multimorbidity are unlikely to be significantly affected by temporal ambiguity. Second, participants without complete NACM data were excluded from the final analyses, which may have introduced selection bias. Third, we focused on 10 common non-AIDS chronic conditions. While this provides useful insight, a broader exploration of multimorbidity among PWH is warranted but requires huge efforts and resources, as PWH are at higher risks for many other NACMs, such as cancer and cardiovascular and metabolic diseases, as compared with the general population. Additionally, women with HIV in our study had longer exposure to antiretroviral therapy and better HIV control as compared with men, which may have influenced the observed interactions between HIV status and sex in relation to multimorbidity.

More than half of PWH globally are women [50], and women with HIV are disproportionately saddled with multiple chronic conditions. However, women remain underrepresented in HIV research and services, especially in the Asian population [51]. Our findings strongly underscore the importance and urgent need for sexually oriented integrative interventions and health services targeting NACM in PWH, with specific priorities to women with HIV.

## Supplementary Material

ofaf046_Supplementary_Data

## Supplementary Data

Supplementary materials are available at *Open Forum Infectious Diseases* online. Consisting of data provided by the authors to benefit the reader, the posted materials are not copyedited and are the sole responsibility of the authors, so questions or comments should be addressed to the corresponding author.

## Notes

***Author contributions*.** N. H. and H. L. proposed the research question and generally supervised the study. X. C. and C. G. contributed to data collection, data analysis, and manuscript drafting. T. W., W. S., S. W., Y. W., T. C., and M. W. contributed to field investigation, sample collection, laboratory work, data management, and data analysis. All authors critically reviewed and edited the manuscript and consented to final publication.

***Data availability*.** All the data in the article or Supplementary materials are free to obtain. Raw data that support the findings of this study are available from the corresponding author upon reasonable request.

***Patient consent statement*.** This study was approved by the institutional review board of the Fudan University School of Public Health, Shanghai, China. All individuals provided written informed consent.

***Role of the funding source*.** The sponsors of the study had no role in study design, data collection, data analysis, data interpretation, or writing of the report. The corresponding author had full access to all the data in the study and had final responsibility for the decision to submit for publication.

***Financial support.*** This work was supported by the Zhejiang Provincial Program for the Cultivation of High-Level Innovative Health Talents (grant 2018-62); the Zhejiang Provincial Natural Science Foundation of China (grant TGY23H260003); the Special Support Program for High Level Talents in Taizhou City (grant TZ2022-2); the Shanghai Municipal Health Commission (grant GWVI-11.1-05); and the Yiwu Research Institute (grant KCF201512) of Fudan University.
