# Supplementary material for: Disproportionate Vulnerability to and Unique Aggregation Pattern of Non-AIDS Comorbidities Among Women With HIV in China
Source: Open Forum Infect Dis. 2025 Jan 29;12(2):ofaf046. doi: 10.1093/ofid/ofaf046 (PMC11811902; doi:10.1093/ofid/ofaf046)
Supplement: ofaf046_Supplementary_Data [file ofaf046_supplementary_data.docx]

**Supplementary materials**

**Table S1.** Prevalence of NACM and multimorbidity in HIV-positive and HIV-negative individuals by sex

**Table S2.** Sex differences on prevalence of multimorbidity between HIV-infected patients and those without HIV infection by age group

**Table S3.** Multivariable analyses of risk factors correlated with NACM burden

**Figure S1.** Prevalence of multimorbidity across four groups (women with HIV, women without HIV, men with HIV, and men without HIV) by characteristics and lifestyles.

**Figure S2.** Proportions of participants with different number of non-AIDS comorbidity (NACM) by HIV status, sex, and age group.

**Figure S3.** Heatmaps of percentage of individuals comprising the most prevalent combinations of multimorbidity among different HIV and sex groups.

**Table S1. Prevalence of NACM and multimorbidity in HIV-positive and HIV-negative individuals by sex**

| **Prevalence, n (%)** | **Women (n = 2060)** | | | | | | **Men (n = 5795)** | | | | | |
| --- | --- | --- | --- | --- | --- | --- | --- | --- | --- | --- | --- | --- |
|  | **18-29y** | **30-39y** | **40-49y** | **50-59y** | **60-69y** | **≥70y** | **18-29y** | **30-39y** | **40-49y** | **50-59y** | **60-69****y** | **≥70y** |
| Hypertension | 12  (3.6) | 34  (7.0) | 76 (16.8) | 167 (38.4) | 153 (55.2) | 53 (73.6) | 117 (10.2) | 221 (18.4) | 407 (29.7) | 422 (44.2) | 414 (56.5) | 238 (62.1) |
| HIV+ | 1  (1.4) | 14 (10.2) | 19 (16.2) | 30 (26.8) | 40 (46.5) | 8  (36.4) | 30  (7.3) | 53 (14.1) | 104 (21.9) | 91 (29.2) | 98 (41.4) | 61 (50.8) |
| HIV- | 11  (4.2) | 20  (5.7) | 57  (17.0) | 137 (42.4) | 113 (59.2) | 45  (90.0) | 87 (11.7) | 168 (20.3) | 303 (33.9) | 331 (51.6) | 316 (63.7) | 177 (67.3) |
| Diabetes | 7  (2.1) | 10  (2.1) | 22  (4.9) | 45 (10.3) | 47 (17.0) | 17 (23.6) | 17  (1.5) | 44  (3.7) | 136 (9.9) | 162 (17.0) | 165 (22.5) | 89 (23.2) |
| HIV+ | 2  (2.7) | 2  (1.5) | 9  (7.7) | 9  (8.0) | 12  (14.0) | 5  (22.7) | 5  (1.2) | 10  (2.7) | 36  (7.6) | 34 (10.9) | 39 (16.5) | 16 (13.3) |
| HIV- | 5  (1.9) | 8  (2.3) | 13  (3.9) | 36 (11.1) | 35 (18.3) | 12  (24.0) | 12  (1.6) | 34  (4.1) | 100 (11.2) | 128 (19.9) | 126 (25.4) | 73 (27.8) |
| Dyslipidemia | 131 (38.8) | 197 (40.6) | 266 (58.7) | 307 (70.6) | 223 (80.5) | 57 (79.2) | 570 (49.5) | 869 (72.2) | 987 (72.1) | 669 (70.1) | 494 (67.4) | 234 (61.1) |
| HIV+ | 29 (39.7) | 59 (43.1) | 68 (58.1) | 71 (63.4) | 54 (62.8) | 20 (90.9) | 185 (45.0) | 234 (62.1) | 300 (63.2) | 178 (57.1) | 125 (52.7) | 69 (57.5) |
| HIV- | 102 (38.5) | 138 (39.7) | 198 (58.9) | 236 (73.1) | 169 (88.5) | 37  (74.0) | 385 (52.0) | 635 (76.8) | 687 (76.8) | 491 (76.5) | 369 (74.4) | 165 (62.7) |
| SCA | 15  (4.4) | 36  (7.4) | 66 (14.6) | 149 (34.3) | 166 (59.9) | 55 (76.4) | 82  (7.1) | 173 (14.4) | 407 (29.7) | 462 (48.4) | 500 (68.2) | 298 (77.8) |
| HIV+ | 9  (12.3) | 22 (16.1) | 21 (17.9) | 51 (45.5) | 61 (70.9) | 17 (77.3) | 54 (13.1) | 73 (19.4) | 163 (34.3) | 163 (52.2) | 158 (66.7) | 95 (79.2) |
| HIV- | 6  (2.3) | 14  (4.0) | 45 (13.4) | 98 (30.3) | 105 (55.0) | 38  (76.0) | 28  (3.8) | 100 (12.1) | 244 (27.3) | 299 (46.6) | 342 (69.0) | 203 (77.2) |
| RI | 28  (8.3) | 49 (10.1) | 115 (25.4) | 197 (45.3) | 177 (63.9) | 68 (94.4) | 79  (6.9) | 184 (15.3) | 341 (24.9) | 375 (39.3) | 424 (57.8) | 329 (85.9) |
| HIV+ | 12 (16.4) | 19 (13.9) | 34 (29.1) | 49 (43.8) | 46 (53.5) | 21 (95.5) | 21  (5.1) | 49  (13.0) | 105 (22.1) | 122 (39.1) | 120 (50.6) | 103 (85.8) |
| HIV- | 16  (6.0) | 30  (8.6) | 81 (24.1) | 148 (45.8) | 131 (68.6) | 47  (94.0) | 58  (7.8) | 135 (16.3) | 236 (26.4) | 253 (39.4) | 304 (61.3) | 226 (85.9) |
| NCI | 11  (3.3) | 13  (2.7) | 30  (6.6) | 46 (10.6) | 49 (17.7) | 25 (34.7) | 30  (2.6) | 43  (3.6) | 58  (4.2) | 67  (7.0) | 103 (14.1) | 98 (25.6) |
| HIV+ | 9  (12.3) | 10  (7.3) | 20 (17.1) | 28  (25.0) | 24 (27.9) | 10 (45.5) | 20  (4.9) | 25  (6.6) | 35  (7.4) | 35 (11.2) | 69 (29.1) | 47 (39.2) |
| HIV- | 2  (0.8) | 3  (0.9) | 10  (3.0) | 18  (5.6) | 25 (13.1) | 15  (30.0) | 10  (1.3) | 18  (2.2) | 23  (2.6) | 32  (5.0) | 34  (6.9) | 51 (19.4) |
| Depression | 74 (21.9) | 133 (27.4) | 111 (24.5) | 120 (27.6) | 92 (33.2) | 22 (30.6) | 232 (20.1) | 236 (19.6) | 306 (22.4) | 259 (27.1) | 214 (29.2) | 109 (28.5) |
| HIV+ | 30 (41.1) | 61 (44.5) | 56 (47.9) | 53 (47.3) | 42 (48.8) | 9  (40.9) | 152 (37.0) | 136 (36.1) | 177 (37.3) | 119 (38.1) | 118 (49.8) | 53 (44.2) |
| HIV- | 44 (16.6) | 72 (20.7) | 55 (16.4) | 67 (20.7) | 50 (26.2) | 13  (26.0) | 80 (10.8) | 100 (12.1) | 129 (14.4) | 140 (21.8) | 96 (19.4) | 56 (21.3) |
| Anemia | 31  (9.2) | 61 (12.6) | 73 (16.1) | 52 (12.0) | 33 (11.9) | 17 (23.6) | 32  (2.8) | 35  (2.9) | 77  (5.6) | 68  (7.1) | 71  (9.7) | 53 (13.8) |
| HIV+ | 10 (13.7) | 19 (13.9) | 27 (23.1) | 16 (14.3) | 12  (14.0) | 6  (27.3) | 22  (5.4) | 23  (6.1) | 50 (10.5) | 42 (13.5) | 44 (18.6) | 36  (30.0) |
| HIV- | 21  (7.9) | 42 (12.1) | 46 (13.7) | 36 (11.1) | 21  (11.0) | 11  (22.0) | 10  (1.3) | 12  (1.5) | 27  (3.0) | 26  (4.0) | 27  (5.4) | 17  (6.5) |
| ALF | 13  (3.8) | 19  (3.9) | 23  (5.1) | 20  (4.6) | 7  (2.5) | 2  (2.8) | 74  (6.4) | 92  (7.6) | 65  (4.7) | 36  (3.8) | 17  (2.3) | 4  (1.0) |
| HIV+ | 6  (8.2) | 10  (7.3) | 13 (11.1) | 8  (7.1) | 3  (3.5) | 1  (4.5) | 22  (5.4) | 27  (7.2) | 32  (6.7) | 13  (4.2) | 8  (3.4) | 1  (0.8) |
| HIV- | 7  (2.6) | 9  (2.6) | 10  (3.0) | 12  (3.7) | 4  (2.1) | 1  (2.0) | 52  (7.0) | 65  (7.9) | 33  (3.7) | 23  (3.6) | 9  (1.8) | 3  (1.1) |
| EA | 102 (30.2) | 149 (30.7) | 139 (30.7) | 155 (35.6) | 140 (50.5) | 41 (56.9) | 408 (35.4) | 380 (31.6) | 486 (35.5) | 412 (43.2) | 376 (51.3) | 227 (59.3) |
| HIV+ | 28 (38.4) | 50 (36.5) | 41  (35.0) | 45 (40.2) | 44 (51.2) | 12 (54.5) | 165 (40.1) | 138 (36.6) | 153 (32.2) | 134 (42.9) | 112 (47.3) | 61 (50.8) |
| HIV- | 74 (27.9) | 99 (28.4) | 98 (29.2) | 110 (34.1) | 96 (50.3) | 29  (58.0) | 243 (32.8) | 242 (29.3) | 333 (37.2) | 278 (43.3) | 264 (53.2) | 166 (63.1) |
| Multimorbidity | 120 (35.5) | 216 (44.5) | 286 (63.1) | 362 (83.2) | 268 (96.8) | 72 (100.0) | 496 (43.1) | 716 (59.5) | 1008 (73.6) | 835 (87.5) | 690 (94.1) | 377 (98.4) |
| HIV+ | 42 (57.5) | 87 (63.5) | 96 (82.1) | 102 (91.1) | 81 (94.2) | 22 (100.0) | 216 (52.6) | 238 (63.1) | 354 (74.5) | 269 (86.2) | 222 (93.7) | 120 (100.0) |
| HIV- | 78 (29.4) | 129 (37.1) | 190 (56.5) | 260 (80.5) | 187 (97.9) | 50 (100.0) | 280 (37.8) | 478 (57.8) | 654 (73.2) | 566 (88.2) | 468 (94.4) | 257 (97.7) |

Abbreviations: NACM = non-AIDS comorbidities, SCA = Subclinical atherosclerosis, RI = Renal impairment, NCI = Neurocognitive impairment, ALF = Abnormal liver function, EA = Electrocardiographic abnormalities.

**Table S2. Sex differences on prevalence of multimorbidity between HIV-infected patients and those without HIV infection by age group**

| **Age group** | **Multimorbidity (%): HIV+ vs HIV-** | **cOR (95%CI) ^a^** | **cOR (95%CI) for interaction ^b^** | **cRERI (95%CI) ^c^** | **aOR (95%CI) ^a^** | **aOR (95%CI) for interaction ^b^** | **aRERI (95%CI) ^c^** |
| --- | --- | --- | --- | --- | --- | --- | --- |
| Total |  |  | 2.1 (1.7-2.8)^***^ | 0.6 (0.4-0.8)^***^ |  | 1.8 (1.3-2.4)^***^ | 0.5 (0.3-0.7)^***^ |
| Women | 78.6 vs 59.1 | 2.5 (2.0-3.2)^***^ |  |  | 2.7 (2.0-3.6)^***^ |  |  |
| Men | 73.4 vs 70.0 | 1.2 (1.1-1.3)^**^ |  |  | 1.5 (1.3-1.8)^***^ |  |  |
| Age 18-29y |  |  | 1.8 (1.0-3.2) | 0.5 (0.1-1.0)^*^ |  | 1.4 (0.7-2.6) | 0.5 (-0.4,1.3) |
| Women | 57.5 vs 29.4 | 3.2 (1.9-5.5)^***^ |  |  | 2.5 (1.2-5.1)^*^ |  |  |
| Men | 52.6 vs 37.8 | 1.8 (1.4-2.3)^***^ |  |  | 2.3 (1.8-3.1)^***^ |  |  |
| Age 30-39y |  |  | 2.4 (1.5-3.8)^***^ | 0.6 (0.3-0.9)^***^ |  | 2.0 (1.2-3.3)^**^ | 0.5 (0.1-0.8)^**^ |
| Women | 63.5 vs 37.1 | 3.0 (2.0-4.5)^***^ |  |  | 3.2 (2.0-5.3)^***^ |  |  |
| Men | 63.1 vs 57.8 | 1.3 (1.0-1.6) |  |  | 1.4 (1.1-1.9)^*^ |  |  |
| Age 40-49y |  |  | 3.3 (1.8-5.8)^***^ | 0.8 (0.6-1.0)^***^ |  | 2.7 (1.5-4.9)^***^ | 0.7 (0.4-0.9)^***^ |
| Women | 82.1 vs 56.5 | 3.5 (2.1-5.9)^***^ |  |  | 3.3 (1.9-5.9)^***^ |  |  |
| Men | 74.5 vs 73.2 | 1.1 (0.8-1.4) |  |  | 1.3 (1.0-1.8)^*^ |  |  |
| Age 50-59y |  |  | 2.9 (1.3-6.6)^**^ | 0.7 (0.4-1.1)^***^ |  | 2.8 (1.2-6.5)^*^ | 0.6 (0.4-0.9)^***^ |
| Women | 91.1 vs 80.5 | 2.5 (1.2-5.0)^*^ |  |  | 2.7 (1.3-5.8)^*^ |  |  |
| Men | 86.2 vs 88.2 | 0.8 (0.6-1.3) |  |  | 0.9 (0.6-1.5) |  |  |
| Age 60-69y |  |  | 0.4 (0.1-1.7) | -2.0 (-5.9, 1.9) |  | 0.4 (0.1-1.7) | -1.5 (-5.3, 2.3) |
| Women | 94.2 vs 97.9 | 0.3 (0.1-1.3) |  |  | 0.4 (0.1-1.5) |  |  |
| Men | 93.7 vs 94.4 | 0.9 (0.5-1.7) |  |  | 1.0 (0.5-2.0) |  |  |
| Age ≥70y |  |  | NA | NA |  | NA | NA |
| Women | 100.0 vs 100.0 | NA |  |  | NA |  |  |
| Men | 100.0 vs 97.7 | NA |  |  | NA |  |  |

Abbreviations: cOR = crude odds ratio, aOR = adjusted odds ratio, cRERI = crude relative excess risk due to interaction, aRERI = adjusted relative excess risk due to interaction, NA = not applicable.

^a^ OR (95%CI) for the correlation of HIV infection and multimorbidity.

^b^ OR (95%CI) for the correlation of interaction term (HIV and sex) and multimorbidity on multiplicative scale.

^c^ RERI (95%CI) for the correlation of interaction term (HIV and sex) and multimorbidity on additive scale.

^***^ P < 0.001, ^**^ P < 0.01, ^*^ P < 0.05.

**Table S3. Multivariable analyses of risk factors correlated with NACM burden**

| **Risk factor** | **All participants** | |  | **Women** | |  | **Men** | |
| --- | --- | --- | --- | --- | --- | --- | --- | --- |
|  | **β (SE)** | ***P value* ^a^** |  | **β (SE)** | ***P value*** |  | **β (SE)** | ***P value*** |
| HIV (Ref = without HIV) | 0.3 | <0.001 |  | 0.5 | <0.001 |  | 0.2 | <0.001 |
| Women (Ref = Men) | -0.2 | <0.001 |  | NA | NA |  | NA | NA |
| Age group, y |  |  |  |  |  |  |  |  |
| 18-29 | [Reference] |  |  | [Reference] | NA |  | [Reference] | NA |
| 30-39 | 0.3 | <0.001 |  | 0.1 | 0.396 |  | 0.3 | <0.001 |
| 40-49 | 0.6 | <0.001 |  | 0.5 | <0.001 |  | 0.7 | <0.001 |
| 50-59 | 1.3 | <0.001 |  | 1.3 | <0.001 |  | 1.4 | <0.001 |
| 60-69 | 2.1 | <0.001 |  | 2.2 | <0.001 |  | 2.0 | <0.001 |
| ≥70 | 2.7 | <0.001 |  | 3.3 | <0.001 |  | 2.6 | <0.001 |
| Education |  |  |  |  |  |  |  |  |
| Primary school or less | [Reference] |  |  | [Reference] |  |  | [Reference] |  |
| Middle school | -0.1 | 0.013 |  | -0.1 | 0.059 |  | -0.1 | 0.098 |
| High school or above | -0.2 | <0.001 |  | -0.3 | 0.001 |  | -0.2 | <0.001 |
| Current cigarette use (Ref = past or never) | 0.0 | 0.827 |  | -0.0 | 0.953 |  | -0.0 | 0.843 |
| Alcohol use (Ref = No) | -0.0 | 0.567 |  | -0.1 | 0.516 |  | -0.0 | 0.583 |
| Exercise (Ref = No) | 0.1 | 0.031 |  | 0.0 | 0.498 |  | 0.1 | 0.012 |
| BMI, kg/m^2^ |  |  |  |  |  |  |  |  |
| <18.5 | -0.1 | 0.359 |  | -0.0 | 0.925 |  | -0.1 | 0.144 |
| 18.5-23.9 | [Reference] | NA |  | [Reference] | NA |  | [Reference] | NA |
| ≥24 | 0.4 | <0.001 |  | 0.3 | <0.001 |  | 0.4 | <0.001 |
| High WHR (Ref = No) | 0.3 | <0.001 |  | 0.3 | <0.001 |  | 0.3 | <0.001 |

Abbreviations: NACM = non-AIDS comorbidities, BMI = body mass index, WHR = waist-to-hip ratio, NA = not applicable.

^a^ Interaction item (HIV × sex) showed statistically significant (P for interaction <0.001) in the adjusted linear regression model.


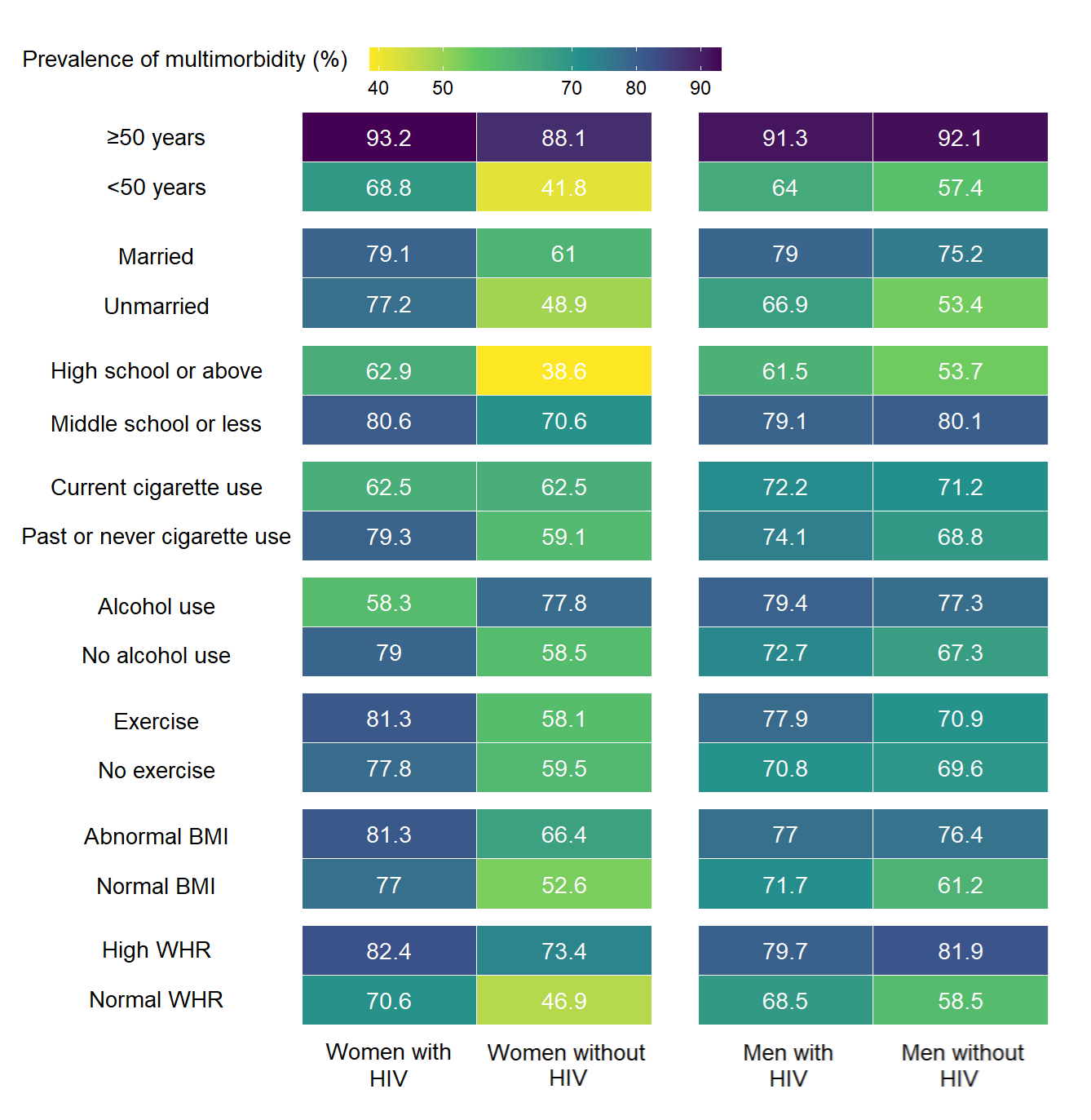
**Figure S1. Prevalence of multimorbidity across four groups (women with HIV, women without HIV, men with HIV, and men without HIV) by characteristics and lifestyles.**


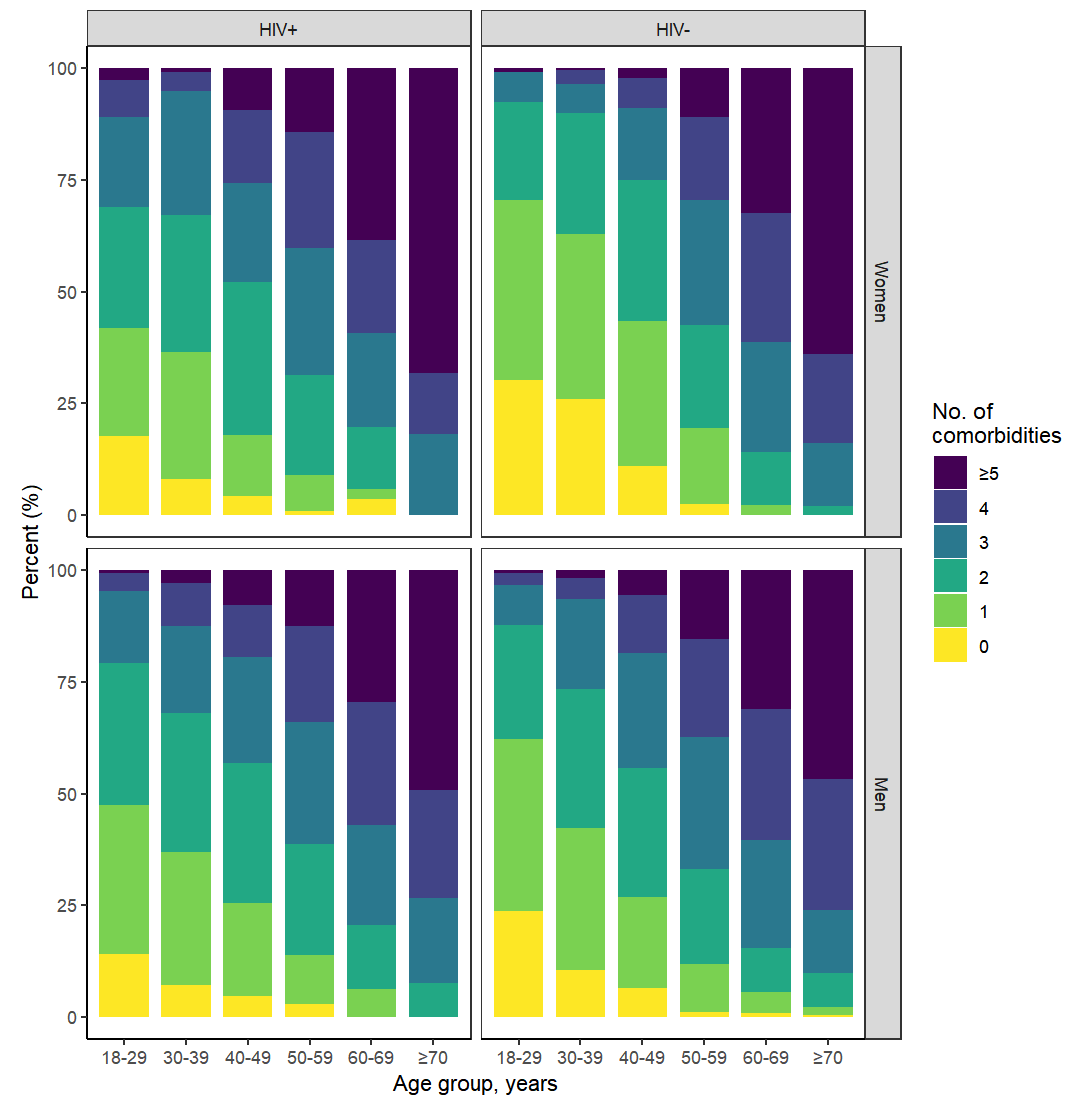


**Figure S2. Proportions of participants with different number of non-AIDS comorbidity (NACM) by HIV status, sex, and age group.**


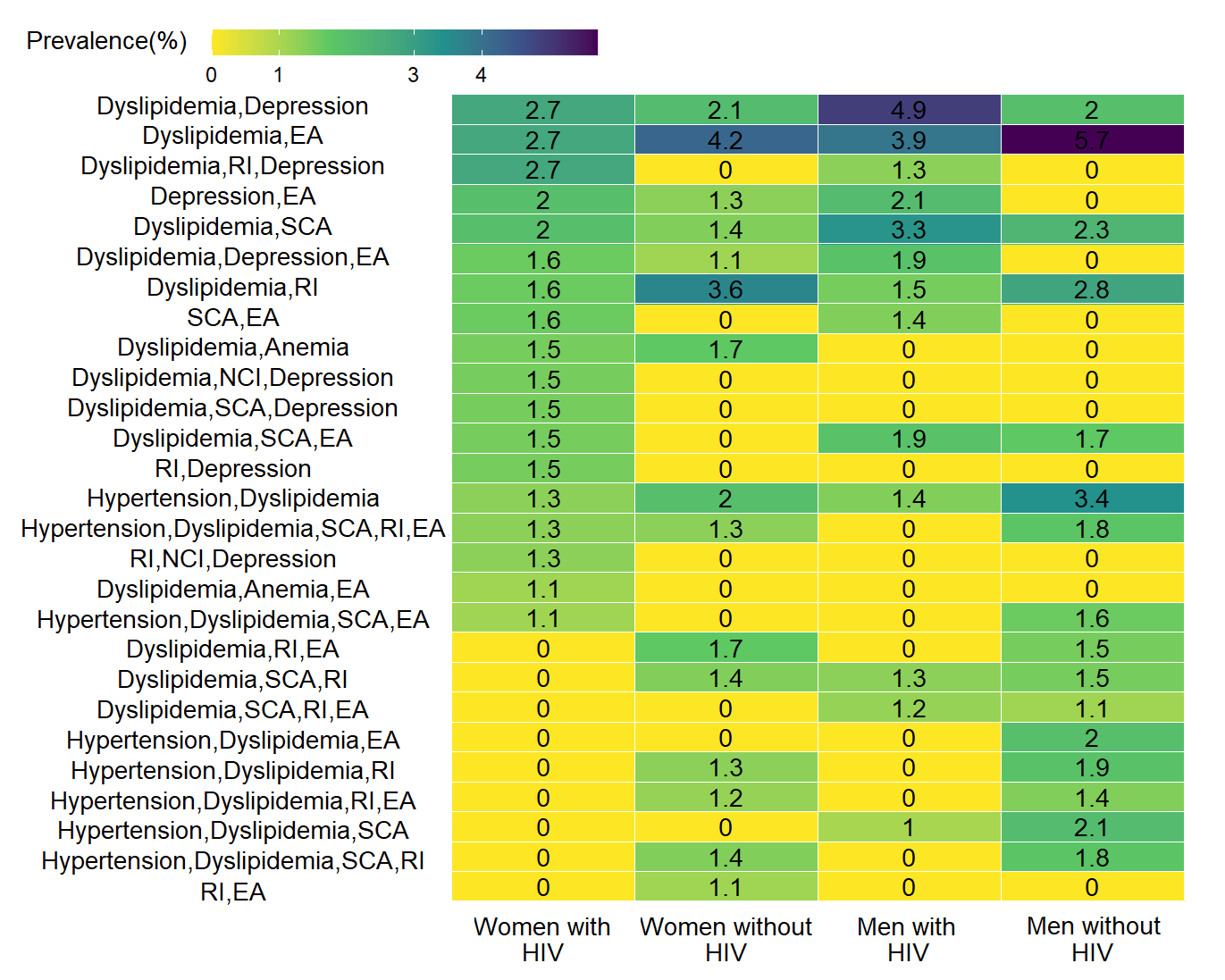


**Figure S3. Heatmaps of percentage of individuals comprising the most prevalent combinations of multimorbidity among different HIV and sex groups.**The value of “0” indicates the prevalence of NACM combination less than 1%. Abbreviations: EA = Electrocardiographic abnormalities, RI = Renal impairment, SCA = Subclinical atherosclerosis, NCI = neurocognitive impairment.
